# Intelligent energy aware optimization protocol for vehicular adhoc networks

**DOI:** 10.1038/s41598-023-35042-6

**Published:** 2023-06-03

**Authors:** Mohamed Elhoseny, Ibrahim M. El-Hasnony, Zahraa Tarek

**Affiliations:** Faculty of Computers and Information, Mansoura University, Mansoura, Egypt

**Keywords:** Energy science and technology, Engineering

## Abstract

Vehicular adhoc network (VANET) plays a vital role in smart transportation. VANET includes a set of vehicles that communicate with one another via wireless links. The vehicular communication in VANET necessitates an intelligent clustering protocol to maximize energy efficiency. Since energy acts as an essential factor in the design of VANET, energy-aware clustering protocols depending upon metaheuristic optimization algorithms are required to be developed. This study introduces an intelligent energy-aware oppositional chaos game optimization-based clustering (IEAOCGO-C) protocol for VANET. The presented IEAOCGO-C technique aims to select cluster heads (CHs) in the network proficiently. The proposed IEAOCGO-C model constructs clusters based on oppositional-based learning (OBL) with the chaos game optimization (CGO) algorithm to improve efficiency. Besides, it computes a fitness function involving five parameters, namely throughput (THRPT), packet delivery ratio (PDR), network lifetime (NLT), end to end delay (ETED) and energy consumption (ECM). The experimental validation of the proposed model is accomplished, and the outcomes are studied in numerous aspects with existing models under several vehicles and measures. The simulation outcomes reported the enhanced performance of the proposed approach over the recent technologies. As a result, it has resulted in maximal NLT (4480), minimal ECM (65.6), maximal THRPT (81.6), maximal PDR (84.5), and minimal ETED (6.7) as average values over the other methods under all vehicle numbers.

## Introduction

Interested vehicles in a vehicular adhoc network (VANET) are equipped with a remote handset that allows them to exchange information with other adjacent vehicles and, if necessary, to route bundles through adjacent vehicles to reach objects that are not in direct interchange range^1^. One-jump availability to the outside foundation isn't required, albeit fixed side of the road units may likewise partake in a VANET. Such a design possibly empowers the making of utilizations going from further developed traffic wellbeing and clog evasion to in-vehicle data and theatre setups^2^. VANETs work in a difficult interchanges’ climate, which to date have restricted the common sense sending of the innovation. VANETs are especially helpless to the secret hub issue; furthermore, they should battle with restricted ghostly data transfer capacity and a profound factor channel affected by both fixed and versatile impediments and obstruction sources^3^. In such a climate, infrastructure-based networks hold a huge benefit over specially appointed organizations: passages permit ideal booking of channel access and appropriation of organization assets in a moderately basic way, at the expense of expecting to convey an enormous number of passageways all through the planned inclusion region^4^. VANET additionally experiences issues, for example, the secret terminal issue, high dormancy for wellbeing data communication, data privacy, broadcast storming issue, nature of administration, quality of service (QoS), parcel directing, blockage control, and asset the executives. To settle these issues, the various leveled structure has been researched in the writing^5^. In a progressive design, at least two close-by vehicles, who have a few normal highlights, participate in a gathering named clustering. It is generally utilized in information mining, and AI^6,7^. VANET consists of stationary or mobile vehicles and RSU units. The main role of the RSU is to collect vehicle data such as vehicle position, direction, speed and other data from the connected sensors. RSU sends a signal to the GPS satellite for sending the gathered data to the nearest server. The server saves and analyses RSU data before sending it to client output apps (computers, phones, tablets, or automobile displays) for thorough statistics and task analysis.

Clustering is a mechanism for aggregating nodes and making the network more robust. With no node consciousness, it eventually runs out of energy, causing network execution difficulties and topology changes. At that moment, a main issue of energy in routing protocol develops, which affects node and link longevity concerns in the network. Clustering is one of the numerous issues that metaheuristic algorithms handle, and they do so because they perform well and have visible practical consequences^8^. A clustering-based optimization strategy was suggested to make V2V communication work better in terms of how much energy it uses. This study introduces an Intelligent Energy Aware Oppositional Chaos Game Optimization-based Clustering (IEAOCGO-C) protocol for VANET. The proposed model aims in the proficient selection of Cluster Heads (CHs) in the network. The contributions of this paper are given as follows:

- The nodes are first initialized and begin to connect with their neighbors.
- Oppositional-Based Learning (OBL) is conducted to determine the CHs and group the nodes into clusters.
- The Chaos Game Optimization algorithm (CGO) is applied to elect effective paths for data transfer using 3 input variables (distance, trust factor, and energy).
- The proposed model constructs clusters based on the integration of OBL with CGO algorithm to improve the total system efficiency.
- The proposed model computes a fitness function involving five parameters namely throughput, packet delivery ratio, network lifetime, end to end delay and energy consumption.
- The obtained results illustrated that the proposed technique is performed better than other existing approaches (FLC, HEPPA, ASC, and LAKAP) under several aspects with different vehicle nodes (20, 40, 60, 80, and 100).

The remainder of the paper is organized as follows: Sect. “Related works” highlights current relevant research and Sect. “The proposed model” provides our recommended approach methods for clustering in VANETs with a detailed view of the proposed model. Section “Oppositional learning” introduces experimental findings and Sect. “Overview of CGO algorithm” concludes the paper by discussing potential future directions.

## Related works

Bidi Ying and Amiya Nayak^9^ presented a smart card (ASC) protocol-based anonymous and lightweight authentication. Compared to standard methods, their protocol might be able to cut communication and processing costs by more than 50%. To demonstrate that their protocol was safe under the premise of the computational Diffie-Hellman issue, a formal security model was created. The simulations also showed that the suggested ASC outperformed others in terms of computational/communication cost, latency, packet loss ratio and so on. Mohammad Wazid et al.^10^ presented a novel lightweight decentralized authentication and key agreement technique for VANETs. The extensive formal and informal security study revealed that the suggested approach could defend against a variety of harmful assaults. Furthermore, the ns-2 simulation proved the suggested scheme's viability in a VANET environment. Chen et al.^11^ presented a connectivity predictive enabled dynamic clustering (DC) technique for VANET in urban transportation. The experimental findings demonstrated that the suggested connection prediction (CP) method could realize a reduced error rate than multilayer perceptron and predictive locations-based geographic routing. Compared to greedy perimeter stateless routing, modified distributed routing, and mobility-adaptive clustering-based methods, the proposed routing technique achieved lower end-to-end latency and a higher delivery rate. A. Ram and M. Mishra^12^ proposed a density-connected cluster-based routing (DCCR) protocol. The method preserved connectedness between two subsequent forwarders by taking into account several matrices such as density and relative velocity. When compared to existing techniques, the suggested protocol revealed an improvement in end-to-end latency and packet delivery ratio. A krill herd optimization technique was suggested by Sadrishojaei and others to choose the cluster head nodes and intermediate nodes needed for routing. The suggested method outperforms particle swarm optimization and cuckoo search in terms of network longevity, according to the NS-3 simulation findings. Comparing the suggested method to the present clustering techniques, the overall network lifespan is increased by at least 11.1%^13^.

DREAMgeoOPT, LARgeoOPT, and ZRPgeoOPT are three geocast routing algorithms designed and assessed by Husain et al.^14^ using particle swarm optimization (PSO) technique. Their protocols performed better since the fitness function employed in PSO decreased latency, routing burden, missed packets, and enhanced throughput. Packet delivery ratio was attained in less time because PSO convergence was quick and local maxima. Ravi Kumar and Barani^15^ presented a reputation-based weighted clustering protocol to combat spiteful routing behavior using the Firefly algorithm. Based on the simulation findings, RBCWP-MOFA outperformed MO-PSO and CL-PSO on three metrics: average number of clusters, packet delivery ratio, and cluster packet overhead. Ali Ghaffari^16^ developed a hybrid opportunistic and location-based routing protocol in VANETs taking into account some factors such as node position, node density and connection quality. The suggested approach selects ideal candidate nodes and assigns suitable priority for data transmission based on the recommended technique. In addition, the suggested technique identified and eliminated expired nodes from the routing process. The simulation findings showed that performance improved in terms of end-to-end latency, throughput, and packet delivery rate. R. Ramamoorthy and M. Thangavelu^17^ presented an Enhanced Hybrid Ant Colony Optimization Routing Protocol (EHACORP) with two stages to increase routing efficiency by utilizing the shortest path. According to the simulation results, EHACORP outperforms ant colony optimization routing algorithm (ARA), Ad hoc on-demand distance vector (AODV), Fuzzy based ant colony optimization (F-ANT), and AntNet routing protocols in terms of throughput, packet delivery rate, routing overhead, end-to-end delay, and packet loss rate. Ubaidullah Rajput et al.^18^ provided a method for ensuring privacy authentication in a VANET. To overcome the limitations of both the group signature-based and pseudonym-based techniques, they developed a hybrid solution that included the best characteristics of each. They provided numerous attack scenarios that demonstrated the suggested approach's resistance to various security and privacy risks. In terms of packet delivery ratio and end-to-end latency, the findings demonstrated the practicality of their suggested strategy.

According to the previous studies, many forms of clustering and routing protocols with varying factors were used for the development of an energy efficient VANET. But there are some disadvantages in these approaches, as high latency, low scalability, inconsiderable multi-hop communication, unsupported mobile nodes, low QoS, low data security, low throughput, high traffic flow, unsupported heterogeneous networks, and unbalanced energy consumption. So, the proposed approach in this paper aims to calculate an efficient fitness function (FF) for CH selection where the CH selection process is dependent on many overlapping metrics based on five input parameters: throughput, packet delivery ratio, network lifetime, end-to-end latency, and energy consumption. Furthermore, the suggested strategy is applicable to small size, medium scale, and large scale VANETs.

## The proposed model

In this study, a proposed technique has been developed for effective energy utilization in VANET via optimal selection of CHs and construction of clusters.

**Figure 1:**
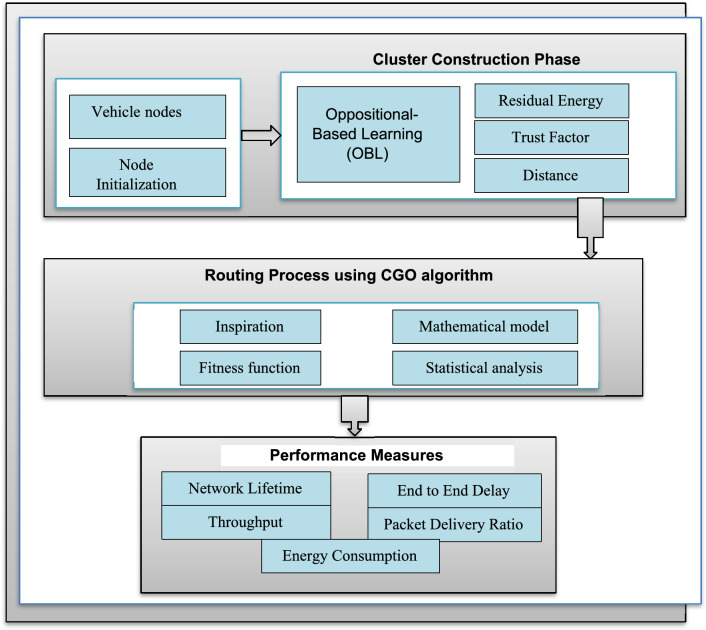
Workflow of the proposed energy efficient optimization IEAOCGO-C technique.

The workflow involved in the current proposed approach is represented in Fig. 1. Consider a VANET with numerous vehicle nodes that are distributed at random in the target environment. The nodes are first initialized and begin to connect with their neighbors. Then, OBL is conducted to determine the CHs and group the nodes into clusters. Next, CGO is applied to elect effective paths for data transfer. Finally, data will be exchanged through intra-cluster and inter-cluster communication procedures.

### Oppositional learning

Oppositional learning is the process of creating opposite solutions to the present solution, thereby deviating the seeking process in the given solution search space^19^. OBL is a population search strategy that generates the opposite of the given solutions and diverts the search process. It is also obvious that applying oppositional search leads to an increased convergence rate towards optimal solutions, as mentioned in Rahnamayan et al.^20^. Algorithm 1 depicts the OBL approach.
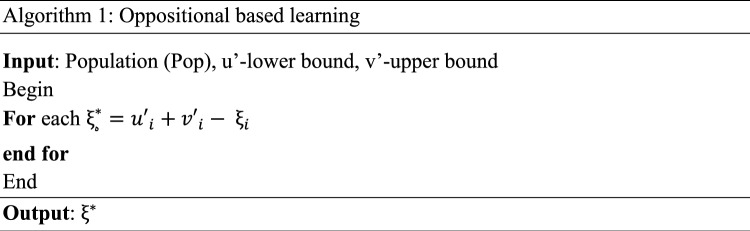


### Overview of CGO algorithm

The CGO method was introduced according to the anticipated rule of chaos model. The basic model of fractal and chaos game was applied to express a mathematical approach for the CGO approach. Because many natural evolutions approach endure a population of solutions which is proceeded through random modification and selection^21^. The CGO method make used of the number of solution candidates (S) at the time of purpose that indicates some appropriate seeds within a Sierpinski triangle. The arithmetical system of this feature is given by:

1 $$S = \begin{array}{*{20}c} {S_{1} } \\ : \\ {S_{n} } \\ \end{array} = \left[ {\begin{array}{*{20}c} {\begin{array}{*{20}c} {S_{1}^{1} } \\ {S_{2}^{1} } \\ \\ {S_{i}^{1} } \\ \end{array} } \\ {S_{n}^{1} } \\ \end{array} \begin{array}{*{20}c} {\begin{array}{*{20}c} {S_{1}^{2} } \\ {S_{2}^{2} } \\ \vdots \\ {S_{i}^{2} } \\ \end{array} } \\ {S_{n}^{2} } \\ \end{array} \begin{array}{*{20}c} {\begin{array}{*{20}c} {S_{1}^{j} } \\ {S_{2}^{j} } \\ \\ {S_{i}^{j} } \\ \end{array} } \\ {S_{n}^{j} } \\ \end{array} \begin{array}{*{20}c} {\begin{array}{*{20}c} \cdots \\ \\ \ddots \\ \cdots \\ \end{array} } \\ \\ \end{array} \begin{array}{*{20}c} {\begin{array}{*{20}c} {S_{1}^{d} } \\ \\ \vdots \\ {S_{n}^{d} } \\ \end{array} } \\ \\ \end{array} } \right]$$

$i = 1, 2 \ldots n. J = 1,2 \ldots . d.$ In which n refers to the count of eligible seed (solution candidate) within the Sierpinski triangle (searching space), and $d$ characterizes the dimension of seeds. The primary place of qualified seeds was described in the searching space:

2 $$S_{1}^{j} \left( 0 \right) = S_{1,min}^{j} + R\left( {S_{1, min}^{j} - S_{1, max}^{j} } \right)$$

while R represents some random number between zero and one. Here is a diagram depicting the first step in the process, which involves planting the primary seed:

3 $$Seed_{i}^{1} = S_{i} + x_{i} *\left( {y_{i} *Global\,best - z_{i} *Mean\,Value} \right)$$

In which $x_{i} , y_{i} , z_{i}$ defines the arbitrary integer of zero or one to demonstrate the possibility of rolling some dice. Then, the schematic performance of described process to the secondary seed is written as:

4 $$Seed_{i}^{2} = Global\,best + x_{i} *\left( {y_{i} *S_{i} - z_{i} *Mean\,Value} \right)$$

In the schematic demonstration of seeds, 3rd and 4th are described as under:

5 $$Seed_{i}^{3} = Mean\,Value + x_{i} *\left( {y_{i} *S_{i} - z_{i} *Global\,best} \right)$$

6 $$Seed_{i}^{4} = S_{i} \left( {S_{i}^{k} = S_{i}^{k} + Rand} \right)$$

In which k stands for the arbitrary integer in the interval of zero and one. The CGO approach, different formulation is projected to $x_{i}$ which controls the movement restriction of seeds.

7 $$x_{i} = \left\{ {\begin{array}{*{20}c} {2*rand} \\ {\left( {\Psi *rand} \right) + 1} \\ {\left( {\Omega *rand} \right) + \sim \Omega } \\ \end{array} } \right.$$

where as $Rand$ signifies the uniformly distributed number in the interval of zero and one. However, $\Psi$ and $\Omega$ are arbitrary integers in the interval [0,1].

For boosting the convergence rate of CGO technique, OBL model was utilized. The quality of primary population solutions with deviation was enhanced by using OBL models. The OBL method explores the entire search space, including the directions that are perpendicular to the ones in which the original solution was found and the ones that were expected to produce the best results^22^. Finally, the OBL techniques assume the suitable solution in all the solutions.

The opposite amount $x$ has been determined as real value on the interval $x \in \left[ {lb, ub} \right]$. The opposite amount of $x$ has demonstrated as $\tilde{x}$ and utilized to calculate the value:

8 $$\tilde{x} = lb + ub - x$$

The abovementioned equation is normalizing for applying from a searching space with several dimensional. Thus, to normalized, all the searching agents and the equivalent opposite places are determined utilizing in Eqs. (9,10,11):

9 $$x = \left[ {x_{1} ,{ }x_{2} ,{ }x_{3} , \ldots x_{D} } \right]$$

10 $$\tilde{x} = \left[ {\tilde{x}_{1} ,\tilde{x}_{2} ,\tilde{x}_{3} , \ldots ,\tilde{x}_{D} } \right]$$

The value of all individual’s components in $\tilde{x}$ is calculated as:

11 $$\tilde{x}_{j} = lb_{j} + ub_{j} - x_{j}\,where\,j = 1,2,3, \ldots , D$$

At this point, the FF is $f\left( . \right)$. After the fitness value $f\left( {\tilde{x}} \right)$ of opposite solution exceed $f\left( x \right)$ of the real solution $x$, then $x = \tilde{x}$; else $x = x$.

The procedure comprised in the CGO algorithm is listed below.

- The population initiate X as $x_{i}$ *where* $\left( {i = 1,2, \ldots ,{ }n} \right)$ .
- Calculate the opposite place of individuals OX as $\tilde{x}_{i}$ *whereas* $\left( {i = 1,2, \ldots ,{ }n} \right)$.
- Choose the $n$ fittest individuals in $\left\{ {X \cup OX} \right\}$ and refer to the population initialization of CGO technique.

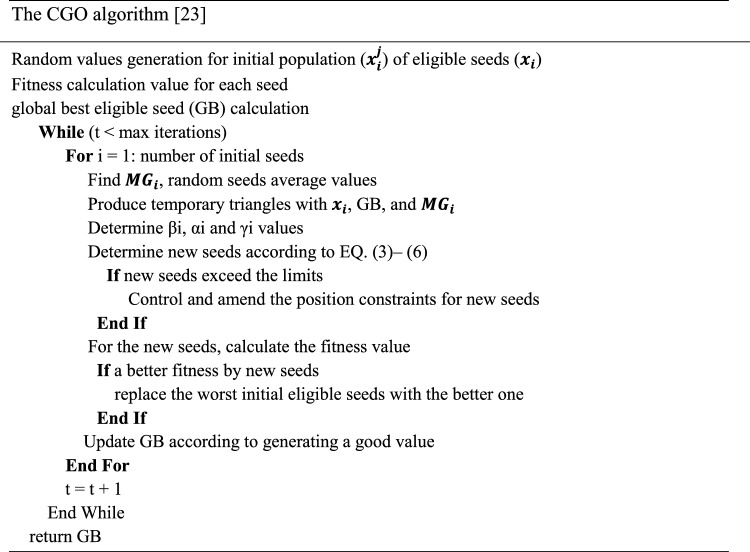


### Design of IEAOCGO-C technique

**Figure 2:**
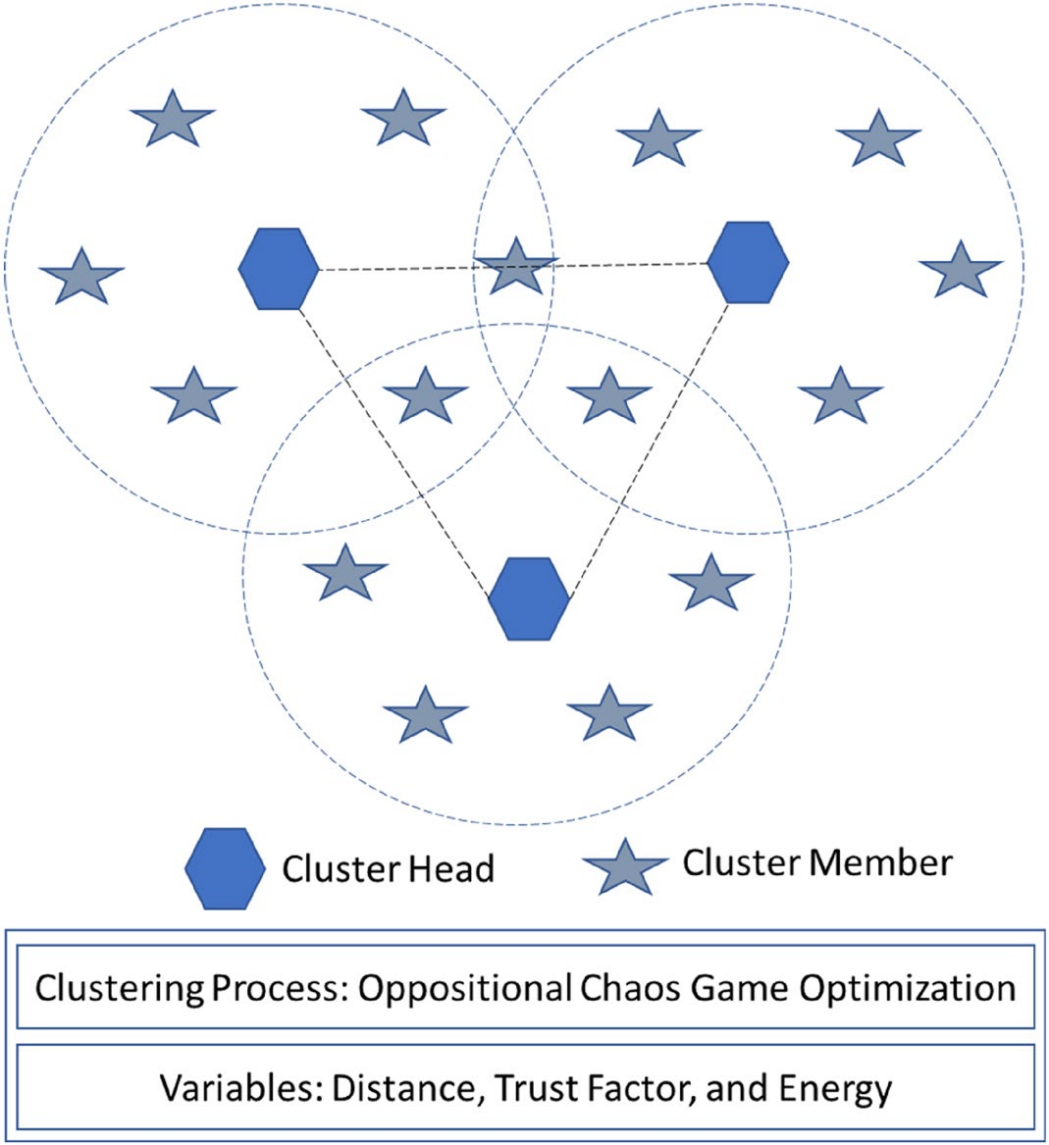
Clustering process.

The proposed technique gets FF with utilize of five input parameters as throughput, packet delivery ratio, end to end delay, network lifetime, and energy to CH election. To improve the efficiency of the proposed model, the CGO algorithm involving three variables, the energy, distance, and trust factor (TF) is utilized for the optimal election of secure CHs. Figure 2 demonstrates the clustering process.

Distance to neighboring nodes is suitable for choosing CH with lesser distance amongst neighboring vehicles^24^. But the intra-cluster transmitting method, sensors vehicle power consumption to CH broadcast. When the neighboring vehicle distance is diminished, then the power of intra- cluster broadcast is also minimalized^25^.

12 $$f_{1} = \mathop \sum \limits_{j = 1}^{m} \frac{1}{{l_{j} }}\left( {\mathop \sum \limits_{i = 1}^{{l_{j} }} dis\left( {CH_{j} ,s_{i} } \right)} \right)$$

where $dis\left( {CH_{j} ,s_{i} } \right)$ is defined as the distance between a cluster head $CH_{j}$ and sensor node $s_{i}$, and m is number of nodes in the network.

Initially, the whole vehicle has been described that TF is one. The value of TF is reduced by abnormal forecasting element when the vehicle processes the anomalous task and vehicle is named as malicious vehicle^26^.

13 $$f_{2} = \mathop \sum \limits_{j = 1}^{m} \frac{1}{m}\left( {TF_{j} } \right)$$

Energy indicates amount of power consumption namely $CHs$ to residual energy (RE) of $CHs$. When the CH employs less power usage as procedure, sense, and transmission method also with maximal RE is gathered as minimal energy ratio. Thus, with a lesser energy ratio, the CH election improves more possibility.

14 $$f_{3} = \mathop \sum \limits_{j = 1}^{m} \frac{{E_{c} \left( {CH_{j} } \right)}}{{E_{R} \left( {CH_{j} } \right)}}$$

where $E_{c} \left( {CH_{j} } \right)$ is the consumed energy of $CH_{j}$, and $E_{R} \left( {CH_{j} } \right)$ is the residual energy of $CH_{j}$. It is important to diminish the linear integration of the main function. Thus, the potential energy function is applied by:

15 $$Minimize\,Potiential\,energy\,function = \alpha_{1} \times f_{1} + \alpha_{2} \times f_{2} + \alpha_{3} \times f_{3}$$

where $$\alpha_{1} + \alpha_{2} + \alpha_{3} = 1, \alpha_{2} \ge \left( {\alpha_{1} + \alpha_{3} } \right). Also 0 < f_{1} , f_{2} ,f_{3} < 1.$$

**Figure 3:**
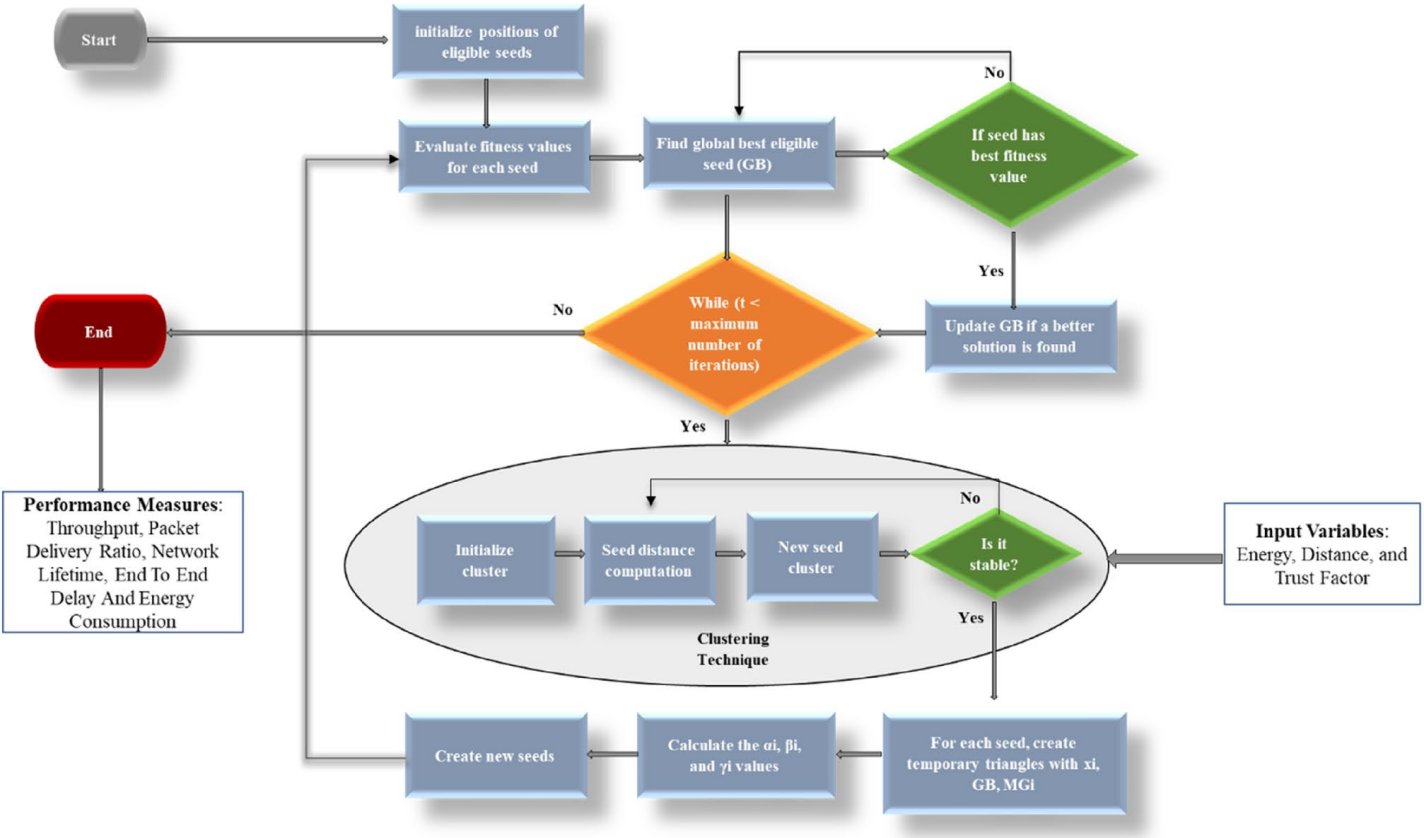
Flowchart of the proposed model.

The potential energy (PE) should be minimized to improve the CH choice. We summarize the process of the proposed Technique in the flowchart of Fig. 3.

## Performance validation

The performance verification of the suggested approach is covered in this section. We examine different grid sizes in terms of network lifetime, throughput, packet delivery ratio, end-to-end delay, and energy consumption. The simulations are run using Network Simulator-2 (NS-2) utilizing the vehicle mobility created by the simulation of urban mobility (SUMO). Even though ns-3 has been entirely redesigned on a new foundation, its predecessor, ns-2, is still widely used and deserves to be mentioned as a separate tool. The most recent version of ns-2 was published in 2011. For this reason, ns-2 uses a method known as discrete-event simulation. Both the definition of the simulated components and the definition of the simulation management and setup are treated as independent tasks in ns-2^27,28^. Microsoft Excel 2016 and MATLAB were utilized throughout the process of generating all the plots and statistics, while figures were generated by Microsoft PowerPoint and Adobe Photoshop.

According to Table 1, the simulator's settings included the number of nodes, the maximum acceleration of the vehicle, the maximum of vehicle speed, the maximum deceleration, RSU Coverage, number of RSUs, and data packet size.

**Table 1:** Parameters settings.

Parameter	Value
Number of nodes	100
Max. acceleration	2.6 m/s^2^
Max. vehicle speed	33 m/s
Max. of deceleration	4.5 m/s^2^
RSU coverage	1 km
Number of RSUs	10
Data packet size	1 kb

In this section, the proposed technique is compared with different techniques, namely, fuzzy logic controller (FLC), lightweight authentication and key agreement protocol (LAKAP), authentication scheme smart card (ASC), and hybrid method for a privacy-preserving authentication approach (HEPPA). The comparison is performed according to the five mentioned performance parameters^26^: Throughput (THRPT), Packet Delivery Ratio (PDR), Network Lifetime (NLT), End to End Delay (ETED), and Energy Consumption (ECM). Table 2 provides a comprehensive comparison study of the proposed model with existing models under several measures and vehicles numbers (20, 40, 60, 80, and 100).

**Table 2:** Comparative analysis of IEAOCGO-C technique with existing models.

No. of vehicles	IEAOCGO-C	FLC Alg	HEPPA Alg	ASC Alg	LAKAP Alg
NLT (rounds)					
20	5000	4600	4600	4100	4000
40	4700	4400	4000	3800	3600
60	4500	3900	3900	3700	3600
80	4200	3800	3400	3300	3100
100	4000	3500	3100	3300	3100
ECM (mJ)					
20	30.96	34.11	43.05	49.87	59.56
40	51.68	61.65	67.72	72.84	82.52
60	70.19	81.23	94.90	96.05	117.72
80	84.91	96.17	112.87	120.64	129.24
100	90.33	105.12	138.19	155.54	175.37
THRPT (kbps)					
20	70.91	63.01	62.90	53.29	51.22
40	77.67	69.53	68.24	60.91	57.71
60	82.57	73.46	70.50	66.46	60.78
80	87.60	78.40	75.71	70.06	66.55
100	89.10	80.01	77.86	76.08	70.36
PDR (%)					
20	99.38	95.89	92.55	90.35	78.04
40	89.36	84.00	80.18	76.69	67.78
60	83.40	75.49	71.51	68.85	59.24
80	76.07	69.34	64.82	56.89	52.53
100	74.21	65.04	59.10	51.24	44.53
ETED (ms)					
20	6.06	8.95	9.37	9.46	12.14
40	6.16	9.67	9.47	9.96	12.14
60	6.44	9.40	10.56	10.92	12.97
80	7.28	9.92	10.99	11.29	13.54
100	7.79	10.85	11.29	11.88	14.19

**Figure 4:**
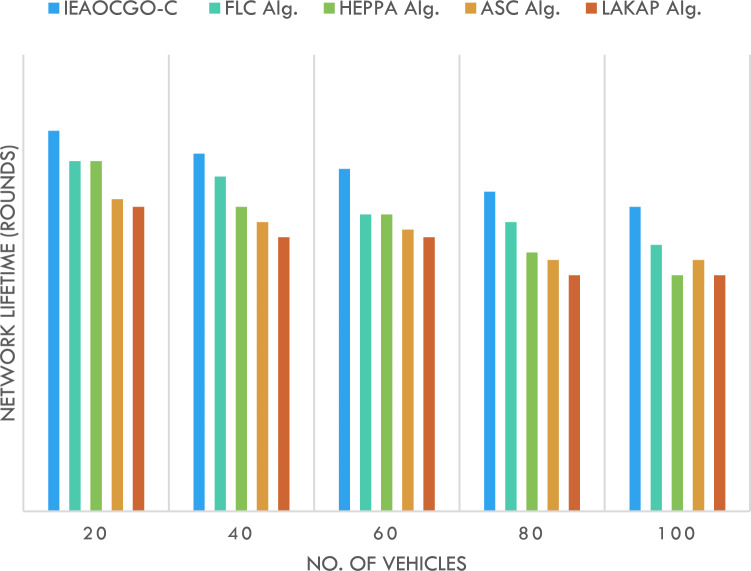
NLT analysis of the proposed technique with recent approaches.

**Figure 5:**
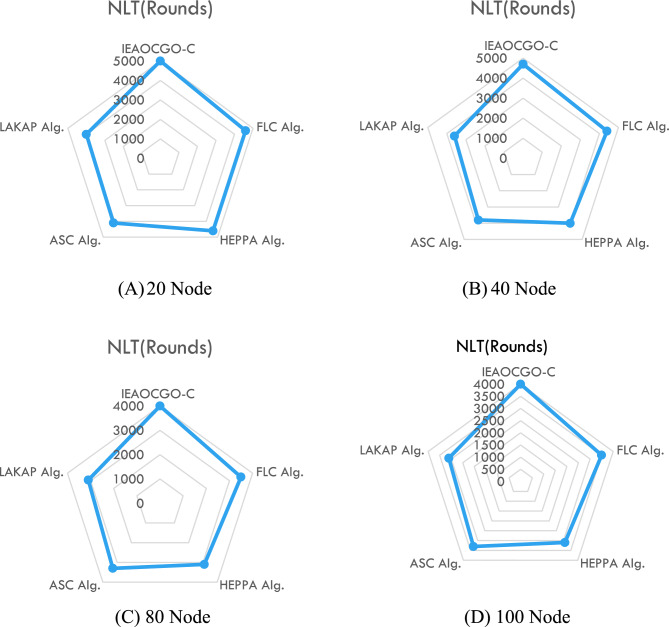
IEAOCGO-C comparison with recent approaches using different vehicles number ((**A**) 20 Node, (**B**) 40 Node, (**C**) 80 Node and (**D**) 100 Node).

Figure 4 reports NLT examination of the proposed technique with recent approaches. It is indicated that the proposed model has resulted in maximum NLT over the other methods under all vehicles. For example, with 20 vehicles, the proposed model has obtained a high NLT of 5000 rounds whereas the FLC technique, HEPPA approach, ASC methodology, and LAKAP algorithm have accomplished low NLT of 4600, 4600, 4100, and 4000 rounds, respectively. Simultaneously, with 100 vehicles, the proposed technique has attained a high NLT of 4000 rounds while the FLC technique, HEPPA approach, ASC methodology, and LAKAP algorithm have accomplished low NLT of 3500, 3100, 3300, and 3100 rounds. The detailed comparison for different nodes number is displayed in Fig. 5.

**Figure 6:**
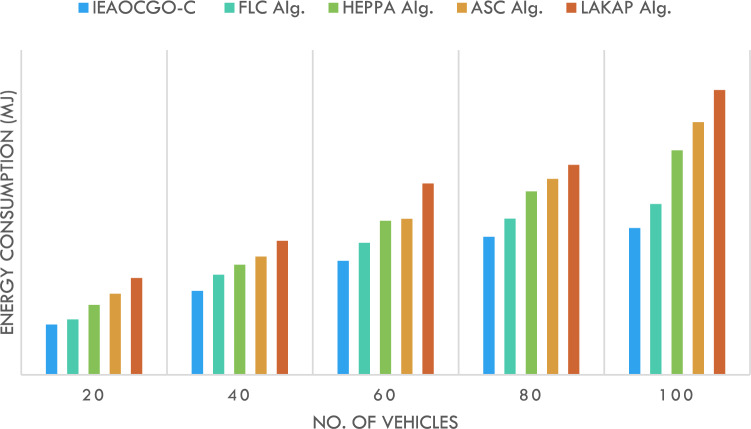
ECM analysis of the proposed technique with recent approaches.

Figure 6 demonstrates the ECM inspection of the proposed approach with existing methods. The results represented the enhanced outcomes of the proposed technique over the other techniques under all vehicle counts. For instance, with 20 vehicles, the proposed method has offered minimal ECM of 30.96 mJ whereas the FLC technique, HEPPA approach, ASC methodology, and LAKAP algorithm have resulted in maximum ECM of 34.11 mJ, 43.05 mJ, 49.87 mJ, and 59.56 mJ respectively. Moreover, with 100 vehicles, the proposed approach has presented minimal ECM of 90.33 mJ while the FLC technique, HEPPA approach, ASC methodology, and LAKAP algorithm have resulted in maximal ECM of 105.12 mJ, 138.19 mJ, 155.54 mJ, and 175.37 mJ correspondingly.

**Figure 7:**
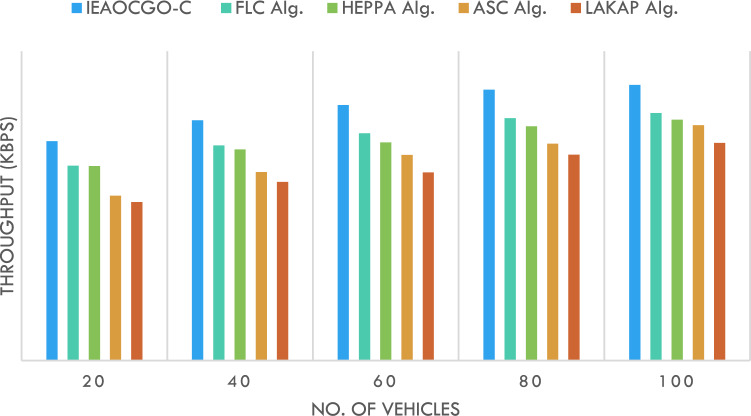
Throughput analysis of IEAOCGO-C technique with recent approaches.

Figure 7 reports the THRPT examination of the proposed technique with current algorithms. It is indicated that the proposed approach has resulted in maximal THRPT over another method under each vehicle. For instance, with 20 vehicles, the proposed approach has gained a high THRPT of 70.91kbps while the FLC technique, HEPPA approach, ASC methodology, and LAKAP algorithm have achieved low THRPT of 63.01kbps, 62.90kbps, 53.29kbps, and 51.22kbps respectively. Simultaneously, with 100 vehicles, the proposed approach has gained a high THRPT of 89.10kbps while the FLC technique, HEPPA approach, ASC methodology, and LAKAP algorithm have accomplished low THRPT of 80.01kbps, 77.86kbps, 76.08kbps, and 70.36kbps correspondingly.

**Figure 8:**
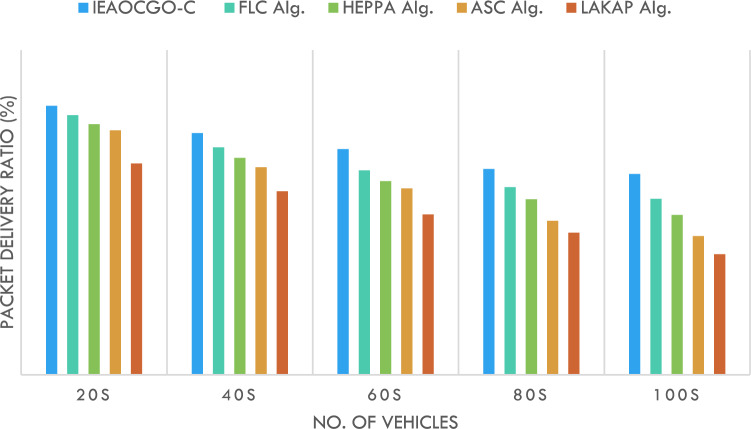
PDR analysis of IEAOCGO-C technique with existing algorithms.

Figure 8 reports the PDR examination of the proposed technique with recent approaches. It is indicated that the proposed technique has resulted in maximal PDR over another method under each vehicle. For instance, with 20 vehicles, the proposed model has attained a high PDR of 99.38% while the FLC technique, HEPPA approach, ASC methodology, and LAKAP algorithm have accomplished low PDR of 95.89%, 92.55%, 90.35%, and 78.04% respectively. Simultaneously, with 100 vehicles, the proposed technique has attained a high PDR of 74.21% while the FLC technique, HEPPA approach, ASC methodology, and LAKAP algorithm have accomplished low PDR of 65.04%, 59.10%, 51.24%, and 44.53% correspondingly.

**Figure 9:**
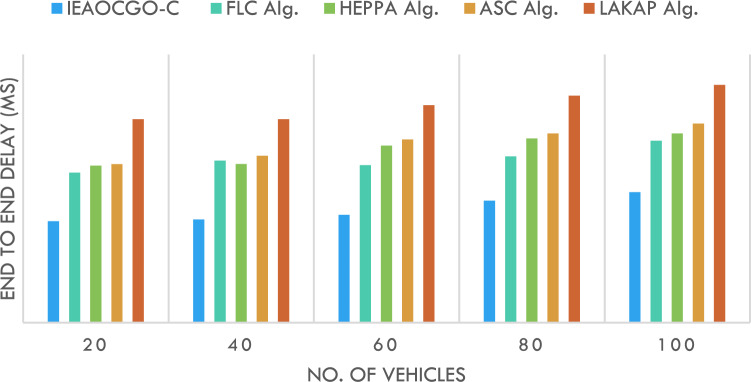
ETED analysis of IEAOCGO-C technique with existing algorithms.

Figure 9 demonstrates the ETED examination of the proposed model with current approaches. The result represents the greater results of the proposed approach over the other algorithms under each vehicle count. For instance, with 20 vehicles, the proposed technique has presented a minimal ETED of 6.06 ms while the FLC technique, HEPPA approach, ASC methodology, and LAKAP algorithm have resulted in maximal ETED of 8.95 ms, 9.37 ms, 9.46 ms, and 12.14 ms correspondingly.

**Figure 10:**
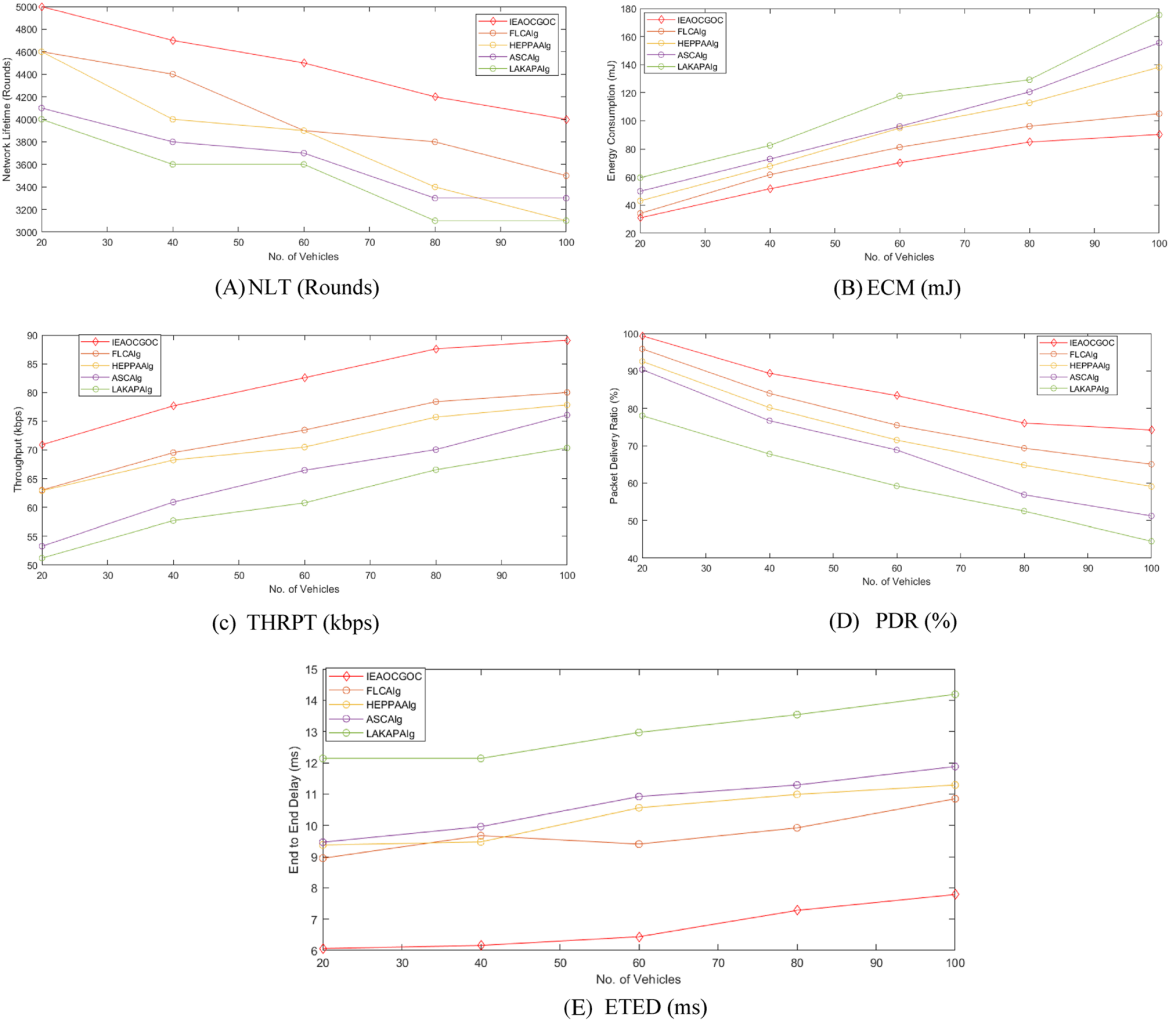
An entire view for the experimental results ((**A**) NLT (**B**) ECM (**C**) THRPT (**D**) PDR (E) ETED).

Furthermore, with 100 vehicles, the proposed technique has presented a minimal ETED of 7.79 ms while the FLC technique, HEPPA approach, ASC methodology, and LAKAP algorithm have resulted in maximal ETED of 10.85 ms, 11.29 ms, 11.88 ms, and 14.19 ms correspondingly. Figure 10 gives the entire view for all comparative results.

It is clear from the thorough experimental study that the proposed model increases throughput, packet delivery ratio, and network lifetime while minimizing delay and energy consumption. The introduction of the CGO method, which involves three variables—the energy, distance, and trust factor—for the best selection of secure CHs, has increased the performance of the suggested model.

## Conclusion

In this study, a new IEAOCGO-C technique has been developed for effective energy utilization in VANET via the optimal selection of CHs and construction of clusters. Several input factors, including residual energy, distance, and trust degree, are used with CGO algorithm. As, secure CHs can be chosen thanks to the node's trust level being included, distance and RE. The proposed technique constructs clusters based on the integration of OBL with CGO algorithm to improve its efficiency. A series of tests were conducted to confirm the findings about the efficacy of the proposed approach compared with existing approaches. The findings were assessed using several metrics, including network lifetime, energy consumption, packet delivery ratio, throughput, and end-to-end latency. The simulation outcomes reported the enhanced performance of the proposed approach over the existing methods as it has resulted in maximal NLT, minimal ECM, maximal THRPT, maximal PDR, and minimal ETED over the other methods under different aspects. In the future, the OCGO algorithm is protracted to the usage of route selection process in VANET.

## Data Availability

No dataset used in the current study. The experimental validation of the proposed model is examined under several vehicle counts using simulators for validations.
